# Supplementary material for: Previously Undescribed Gross HACE1 Deletions as a Cause of Autosomal Recessive Spastic Paraplegia
Source: Genes (Basel). 2022 Nov 23;13(12):2186. doi: 10.3390/genes13122186 (PMC9778407; doi:10.3390/genes13122186)
Supplement: Supplementary file 1 [file genes-13-02186-s001.zip › HACE 1 isoforms_Supplementary Material S2.pdf]

mRNA sequences

	Primers sequences
AAAAAAA	exon 5 nucleotide sequence (canonical transcript)
<u>AAAAAAA</u>	exon 6 nucleotide sequence (canonical transcript)
**<u>AAAAAAA</u>**	exon 7 nucleotide sequence (canonical transcript)
AAAAAAA	exon 8 nucleotide sequence (canonical transcript)

Isoform a (909 aa) = Transcript variant 1 (4575 bp)

AGAAGGCCACGCGAGCCCGGGAGGGACGCGGCGGCGGGGGCTGCAGGAAAGGCGCGAGCAGAGGCGGCGGC
GGGTGTACTGTAGGTGGTCGGTCCGGCAGCAGCCCGGCCCCGGACGCAGGACGTGGCCCCAGGCAGCCCTCGC
AGCTCAGTGCTCTAGCCGGGGCAAGCCCGCGTCTCCGCCTGCTGGACGGGCCCAGGCGAGATGTAGGGCTCTGG
GCGCGGAGGCCGCCGGTGGGGCGGCTGATCGCGGAGGATCGCGGAGGGCGCGCCGAGGATGGAGAGAGCGAT
GGAGCAACTCAACCGCCTGACGCGCTCGCTGCGCCGCGCGCGCACCGTGGAGTTGCCCGAGGATAATGAAACTG
CTGTTTATACATTAATGCCAATGGTTATGGCTGATCAACACAGGTCTGTTTCTGAACTACTATCAAATTCAAAATTT
GATGTCAATTATGCATTCGGACGTGTGAAAAGAAGCTTGCTTCACATTGCAGCAAATTGTGGATCGGTGGAATGCT
TGGTTTTGCTGTTAAAGAAAGGAGCAAATCCTAACTATCAAGATATTTCAGGCTGTACACCCCTTCA**TTTGGCAGC**
AAGAAATGGGCAGAAGAAATGTATGAGTAAATTATTAGAATATAGCGCTGATGTCAACATTTGTAATAATGAAGG
CCTTACAGCAATACATTGGCTGGCTGTGAATGGGCGGACAGAACTACTCCATGACCTTGTGCAGCATGTCAGTGAT
GTTGATGTTGAGGATGCCATGGGGCAGACAGCACTGCATGTTGCCTGCCAGAACGGTCACAAGACG**ACAGTGCA**
GTGCTTGCTAGACAGTGGTGCTGATATTAACAGGCCAAATGTATCAGGAGCAACTCCATTGTACTTTGCTTGCAG
TCATGGTCAGAGAGATACAGCACAGATCCTACTATTACGAGGAGCCAAATATCTGCCAGATAAAAATGGAGTAAC
TCCTCTGGATTTATGTGTACAGGGTGGATATGGAGAGACTTGTGAAGTATTAATTCAATATCACCCGAGGCTTTTT
CAGACTATTATTCAAATGACACAGAATGAAGACCTCCGAGAAAACATGT**TACGGCAAGTTCTGGAGCAT**TTGTCTC
AGCAAAGTGAAAGCCAGTACCTAAAGATTCTAACAAGCCTTGCTGAAGTTGCTACAACAAATGGTCATAAACTGCT
TAGCCTCTCTAGCAATTATGATGCTCAAATGAAGAGCCTTTTAAGGATTGTGAGAATGTTTTGTCACGTCTTTCGAA
TTGGTCCATCCTCCCCCAGTAATGGAATTGATATGGGCTACAATGGGAATAAAACTCCAAGAAGCCAGGTGTTCAA
GCCTCTGGAATTGCTTTGGCACTCGTTAGATGAATGGCTAGTTTTAATAGCCACAGAATTGATGAAAAACAAAAGA
GACTCAACAGAGATCACTTCTATTTTACTGAAACAAAAAGGCCAAGATCAAGATGCTGCTTCCATTCCTCCATTTGA
ACCTCCAGGACCTGGGAGCTATGAAAATCTGTCCACTGGCACAAGGGAATCTAAACCAGATGCTCTTGCAGGGAG
ACAGGAAGCCAGTGCAGATTGTCAGGATGTTATTTCTATGACAGCTAACCGGCTAAGTGCTGTCATTCAAGCTTTT
TACATGTGCTGTTCTTGTCAGATGCCTCCGGGAATGACTTCACCTCGTTTCATTGAATTTGTCTGCAAACATGATGA
AGTTTTAAAATGCTTTGTTAATAGAAATCCCAAAATTATATTTGACCACTTTCACTTTCTCCTTGAATGTCCTGAGTT
GATGTCAAGATTCATGCATATCATAAAAGCACAGCCTTTTAAAGATCGCTGTGAATGGTTCTATGAACATTTGCATT
CAGGACAGCCAGATTCAGATATGGTGCACAGGCCAGTGAATGAAAATGATATCCTGCTGGTTCACAGAGATTCTA
TTTTTAGGAGTAGCTGTGAAGTTGTGTCAAAAGCAAATTGTGCAAAGCTAAAGCAAGGGATTGCTGTACGGTTCC
ATGGAGAAGAAGGCATGGGTCAAGGTGTTGTGCGTGAGTGGTTTGATATTCTGTCCAATGAGATAGTCAATCCTG
ATTATGCATTGTTTACCCAGTCAGCTGATGGAACAACTTTTCAGCCTAATAGCAACTCTTATGTAAATCCTGATCACT
TGAACTATTTTCGGTTTGCTGGGCAGATCTTGGGATTAGCGTTGAACCACAGGCAGCTGGTCAATATTTACTTCAC
ACGATCCTTCTACAAGCACATTCTTGGTATTCCTGTAAATTACCAAGATGTGGCATCCATTGATCCAGAATATGCGA
AAAATTTGCAATGGATTTTAGATAATGATATAAGTGATCTGGGTCTAGAACTAACTTTTTCTGTTGAGACTGATGTG
TTTGGAGCAATGGAAGAGGTGCCTTTGAAACCTGGGGGTGGGAGTATTCTTGTGACACAAAATAATAAAGCGGA
GTACGTCCAGCTTGTTACTGAACTTCGAATGACAAGAGCCATTCAGCCTCAGATCAATGCTTTTTTACAGGGCTTTC
ATATGTTCATTCCACCCTCCCTCATACAGCTTTTTGATGAATATGAATTGGAGCTACTGCTTTCTGGCATGCCAGAA
ATTGATGTGAGTGATTGGATAAAAAATACAGAATACACAAGTGGCTATGAAAGAGAAGATCCAGTTATTCAGTGG
TTCTGGGAAGTTGTAGAAGACATTACTCAAGAGGAGAGAGTTCTTCTCTTACAGTTTGTTACGGGCAGTTCCAGGG
TCCCACATGGTGGGTTTGCTAATATCATGGGTGGAAGTGGATTGCAAAACTTTACAATCGCTGCTGTGCCATATAC
TCCAAATCTTTTACCAACTTCAAGCACATGCATCAACATGCTCAAGTTACCTGAATACCCAAGTAAAGAAATACTCA
AGGACAGACTTCTTGTGGCACTACATTGTGGCAGCTATGGTTACACAATGGCATAATGAAGTCTGGAAAACTCCTC
TGACTACTGATGCACAATTCAGAATGGCAGAAGTAATTTGGGAAAATGTCAACAAAAAAGCAGCCTAAATGCAAC

CCATAGGCAGGGCTGATGCTTCCAATTTATAAAGGATCATCAGGTTTTCTGTTTCTCTCTTTTCCCTTTTATGTTTTCT
CTGTTTGTGATACAATTAGAAAATATAAAATCACAGTAGATTTTATTTTTTAAAATGCTAACTGAAAGTAATAGAGA
CTGTCCTTTTTCATAATTAATTTTATCCAAGATTGTATTAAGGCAAAATCTGATTCTACATTCCACCTCTGCTATGTAA
CTGTCTTGTTAAAAGGGTGTTTTCTCCTAATTTCTGATATATTATATGAGGTCATCCAGCTGGTGTGTTCTTTTGCAT
GTAAACTGCCATTTATATTTTAGAAAACTATTGTATAGAATGGATTTAGATTGTCTATAAAGCCACAAATACGTATT
TTGCCACAGTGTATTCTATATTGCAATGATTTTTTTAGCATTTTAATATTTTAATATATATTGTAAAATTTAGACTGAT
GATACTAACAGTTGATGAAATGACATATAATTTATATATGAAAGCTTACGCTATATTGTATGAATTATTTGCATCTTT
CAGTGGCCAGTTTTCCATATGTATATATTATGGTCTCAATGTTTTTCTTACGCCTCATTTTAATTTATAATGAAGGTA
AAATTAAAATGTATTTTACCACGTTTCTTTTCATTACTTTTATCTGTGAGCTCTGACACATCTGAAAAAGTAATCTGA
TGTGCAAATTATAATTTAAATATGTTAATTTTTTTGCTTCTTAAATTTGCTTTTCATCATTAAAATGTCAAGTTCAAGT
GATATGTGCCTAATATCACTTGGATGTTGGTGGGTTTTTGAATTTTTGGGTGGTTAATCAGTTTTATTTTGAAAAGA
CGTACTTGAATAGTTACAGCATATGTTTGAACAGGAAGTAGGAACATGCATACACGAAGAAATGCTAACGGAAGG
ATTTGTTATGTTTAGGATCTTCCCTTGGAAACTAAAAATAGAATATTAATGACATTACTGTTTGTAGAATGACATAT
GCAGATTTTCTCATAAGCAGTCATTGTGTTTGCCAGTAATGTTTGAGAGACATGTAAGTTGAAAGTTTTGCTAAATT
ATAAAGCTCCTTTAATTCGTTGGTTTTGATTCTCTTATTCTCTTGTCTTTTCTAAATGTTAACAAAATATATCTTAACA
GATTACATGAAATTTAGGAATTATTTAAAAGTTACCATTAGCTCTAAAATTAAGATTCGGATGCTTTATTTATAGTA
ACTGAAGCTAATAATGTTTTATGTTTTGATTTTTTGAAATTTAATTGTAGAAGTCACTGCCTTCTGAGTTTTCAAATA
GATAACCACCTTTAATATTACACTGCTTATAATACTAATGTTTACAGATATGTTTCTGTTTATAACCATATAATACATT
GGCTTTGTCATATTAGTTTTTTTTGCAAGTAGTTATGTAAAAGAGATAGATAATAAAATATTAAATAACTGA

Isoform d (741 aa) = Transcript variant 5 (4307 bp)

AGGCTGGCCGCGGGGCTGGGGGCGGCCAGTGGGGCAGGAGCTCCGGCCGCGTCAAGGTCGGGTGGAAAAACTA
ACTGAAAGCTGAGAAAACGCCCACTTTTACTTTCCAGAGCTGAGAGGCGGCCCCGGGCCCCGAACTCCCCTGGCC
CGACGCTCCCACCCCGGCAGATAATGAAACTGCTGTTTATACATTAATGCCAATGGTTATGGCTGATCAACACAGG
TCTGTTTCTGAACTACTATCAAATTCAAAATTTGATGTCAATTATGCATTCGGACGTGTGAAAAGAAGCTTGCTTCA
CATTGCAGCAAATTGTGGATCGGTGGAATGCTTGGTTTTGCTGTTAAAGAAAGGAGCAAATCCTAACTATCAAGAT
ATTTCAGGCTGTACACCCCTTCA**TTTGGCAGCAAGAAATGGGC**AGAAGAAATGTATGAGTAAATTATTAGAATAT
AGCGCTGATGTCAACATTTGTAATAATGAAGGCCTTACAGCAATACATTGGCTGGCTGTGAATGGGCGGACAGAA
CTACTCCATGACCTTGTGCAGCATGTCAGTGATGTTGATGTTGAGGATGCCATGGGGCAGACAGCACTGCATGTT
GCCTGCCAGAACGGTCACAAGACGTCATGGTCAGAGAGATACAGCACAGATCCTACTATTACGAGGAGCCAAATA
TCTGCCAGATAAAAATGGAGTAACTCCTCTGGATTTATGTGTACAGGGTGGATATGGAGAGACTTGTGAAGTATT
AATTCAATATCACCCGAGGCTTTTTCAGACTATTATTCAAATGACACAGAATGAAGACCTCCGAGAAAACATGT**TA**
CGGCAAGTTCTGGAGCATTTGTCTCAGCAAAGTGAAAGCCAGTACCTAAAGATTCTAACAAGCCTTGCTGAAGTT
GCTACAACAAATGGTCATAAACTGCTTAGCCTCTCTAGCAATTATGATGCTCAAATGAAGAGCCTTTTAAGGATTGT
GAGAATGTTTTGTCACGTCTTTCGAATTGGTCCATCCTCCCCCAGTAATGGAATTGATATGGGCTACAATGGGAAT
AAAACTCCAAGAAGCCAGGTGTTCAAGCCTCTGGAATTGCTTTGGCACTCGTTAGATGAATGGCTAGTTTTAATAG
CCACAGAATTGATGAAAAACAAAAGAGACTCAACAGAGATCACTTCTATTTTACTGAAACAAAAAGGCCAAGATC
AAGATGCTGCTTCCATTCCTCCATTTGAACCTCCAGGACCTGGGAGCTATGAAAATCTGTCCACTGGCACAAGGGA
ATCTAAACCAGATGCTCTTGCAGGGAGACAGGAAGCCAGTGCAGATTGTCAGGATGTTATTTCTATGACAGCTAA
CCGGCTAAGTGCTGTCATTCAAGCTTTTTACATGTGCTGTTCTTGTCAGATGCCTCCGGGAATGACTTCACCTCGTT
TCATTGAATTTGTCTGCAAACATGATGAAGTTTTAAAATGCTTTGTTAATAGAAATCCCAAAATTATATTTGACCACT
TTCACTTTCTCCTTGAATGTCCTGAGTTGATGTCAAGATTCATGCATATCATAAAAGCACAGCCTTTTAAAGATCGCT
GTGAATGGTTCTATGAACATTTGCATTCAGGACAGCCAGATTCAGATATGGTGCACAGGCCAGTGAATGAAAATG
ATATCCTGCTGGTTCACAGAGATTCTATTTTTAGGAGTAGCTGTGAAGTTGTGTCAAAAGCAAATTGTGCAAAGCT
AAAGCAAGGGATTGCTGTACGGTTCCATGGAGAAGAAGGCATGGGTCAAGGTGTTGTGCGTGAGTGGTTTGATA
TTCTGTCCAATGAGATAGTCAATCCTGATTATGCATTGTTTACCCAGTCAGCTGATGGAACAACTTTTCAGCCTAAT
AGCAACTCTTATGTAAATCCTGATCACTTGAACTATTTTCGGTTTGCTGGGCAGATCTTGGGATTAGCGTTGAACCA
CAGGCAGCTGGTCAATATTTACTTCACACGATCCTTCTACAAGCACATTCTTGGTATTCCTGTAAATTACCAAGATG
TGGCATCCATTGATCCAGAATATGCGAAAAATTTGCAATGGATTTTAGATAATGATATAAGTGATCTGGGTCTAGA
ACTAACTTTTTCTGTTGAGACTGATGTGTTTGGAGCAATGGAAGAGGTGCCTTTGAAACCTGGGGGTGGGAGTAT

TCTTGTGACACAAAATAATAAAGCGGAGTACGTCCAGCTTGTTACTGAACTTCGAATGACAAGAGCCATTCAGCCT
CAGATCAATGCTTTTTTACAGGGCTTTCATATGTTCATTCCACCCTCCCTCATACAGCTTTTTGATGAATATGAATTG
GAGCTACTGCTTTCTGGCATGCCAGAAATTGATGTGAGTGATTGGATAAAAAATACAGAATACACAAGTGGCTAT
GAAAGAGAAGATCCAGTTATTCAGTGGTTCTGGGAAGTTGTAGAAGACATTACTCAAGAGGAGAGAGTTCTTCTC
TTACAGTTTGTTACGGGCAGTTCCAGGGTCCCACATGGTGGGTTTGCTAATATCATGGGTGGAAGTGGATTGCAA
AACTTTACAATCGCTGCTGTGCCATATACTCCAAATCTTTTACCAACTTCAAGCACATGCATCAACATGCTCAAGTTA
CCTGAATACCCAAGTAAAGAAATACTCAAGGACAGACTTCTTGTGGCACTACATTGTGGCAGCTATGGTTACACAA
TGGCATAATGAAGTCTGGAAAACTCCTCTGACTACTGATGCACAATTCAGAATGGCAGAAGTAATTTGGGAAAAT
GTCAACAAAAAAGCAGCCTAAATGCAACCCATAGGCAGGGCTGATGCTTCCAATTTATAAAGGATCATCAGGTTTT
CTGTTTCTCTCTTTTCCCTTTTATGTTTTCTCTGTTTGTGATACAATTAGAAAATATAAAATCACAGTAGATTTTATTTT
TTAAAATGCTAACTGAAAGTAATAGAGACTGTCCTTTTTCATAATTAATTTTATCCAAGATTGTATTAAGGCAAAAT
CTGATTCTACATTCCACCTCTGCTATGTAACTGTCTTGTTAAAAGGGTGTTTTCTCCTAATTTCTGATATATTATATGA
GGTCATCCAGCTGGTGTGTTCTTTTGCATGTAAACTGCCATTTATATTTTAGAAAACTATTGTATAGAATGGATTTA
GATTGTCTATAAAGCCACAAATACGTATTTTGCCACAGTGTATTCTATATTGCAATGATTTTTTTAGCATTTTAATAT
TTTAATATATATTGTAAAATTTAGACTGATGATACTAACAGTTGATGAAATGACATATAATTTATATATGAAAGCTT
ACGCTATATTGTATGAATTATTTGCATCTTTCAGTGGCCAGTTTTCCATATGTATATATTATGGTCTCAATGTTTTTCT
TACGCCTCATTTTAATTTATAATGAAGGTAAAATTAAAATGTATTTTACCACGTTTCTTTTCATTACTTTTATCTGTGA
GCTCTGACACATCTGAAAAAGTAATCTGATGTGCAAATTATAATTTAAATATGTTAATTTTTTTGCTTCTTAAATTTG
CTTTTCATCATTAAAATGTCAAGTTCAAGTGATATGTGCCTAATATCACTTGGATGTTGGTGGGTTTTTGAATTTTTG
GGTGGTTAATCAGTTTTATTTTGAAAAGACGTACTTGAATAGTTACAGCATATGTTTGAACAGGAAGTAGGAACAT
GCATACACGAAGAAATGCTAACGGAAGGATTTGTTATGTTTAGGATCTTCCCTTGGAAACTAAAAATAGAATATTA
ATGACATTACTGTTTGTAGAATGACATATGCAGATTTTCTCATAAGCAGTCATTGTGTTTGCCAGTAATGTTTGAGA
GACATGTAAGTTGAAAGTTTTGCTAAATTATAAAGCTCCTTTAATTCGTTGGTTTTGATTCTCTTATTCTCTTGTCTTT
TCTAAATGTTAACAAAATATATCTTAACAGATTACATGAAATTTAGGAATTATTTAAAAGTTACCATTAGCTCTAAA
ATTAAGATTCGGATGCTTTATTTATAGTAACTGAAGCTAATAATGTTTTATGTTTTGATTTTTTGAAATTTAATTGTA
GAAGTCACTGCCTTCTGAGTTTTCAAATAGATAACCACCTTTAATATTACACTGCTTATAATACTAATGTTTACAGAT
ATGTTTCTGTTTATAACCATATAATACATTGGCTTTGTCATATTAGTTTTTTTTGCAAGTAGTTATGTAAAAGAGATA
GATAATAAAATATTAAATAACTGA

Isoform d (741 aa) = transcript variant 10 (4492 bp)

AGAAGGCCACGCGAGCCCGGGAGGGACGCGGCGGCGGGGGCTGCAGGAAAGGCGCGAGCAGAGGCGGCGGC
GGGTGTACTGTAGGTGGTCGGTCCGGCAGCAGCCCGGCCCCCGGACGCAGGACGTGGCCCCAGGCAGCCCTCGC
AGCTCAGTGCTCTAGCCGGGGCAAGCCCGCGTCTCCGCCTGCTGGACGGGCCCAGGCGAGATGTAGGGCTCTGG
GCGCGGAGGCCGCCGGTGGGGCGGCTGATCGCGGAGGATCGCGGAGGGCGCGCCGAGGATGGAGAGAGCGAT
GGAGCAACTCAACCGCCTGACGCGCTCGCTGCGCCGCGCGCGCACCGTGGAGTTGCCCGAGGATAATGAAACTG
CTGTTTATACATTAATGCCAATGGTTATGGCTGATCAACACAGGTCTGTTTCTGAACTACTATCAAATTCAAAATTT
GATGTCAATTATGCATTCGGACGTGTGAAAAGAAGCTTGCTTCACATTGCAGCAAATTGTGGATCGGTGGAATGCT
TGGTTTTGCTGTTAAAGAAAGGAGCAAATCCTAACTATCAAGATATTTCAGGCTGTACACCCCTTCA**TTTGGCAGC**
AAGAAATGGGCAGAAGAAATGTATGAGTAAATTATTAGAATATAGCGCTGATGTCAACATTTGTAATAATGAAGG
CCTTACAGCAATACATTGGCTGGCTGTGAATGGGCGGACAGAACTACTCCATGACCTTGTGCAGCATGTCAGTGAT
GTTGATGTTGAGGATGCCATGGGGCAGACAGCACTGCATGTTGCCTGCCAGAACGGTCACAAGACGTCATGGTCA
GAGAGATACAGCACAGATCCTACTATTACGAGGAGCCAAATATCTGCCAGATAAAAATGGAGTAACTCCTCTGGA
TTTATGTGTACAGGGTGGATATGGAGAGACTTGTGAAGTATTAATTCAATATCACCCGAGGCTTTTTCAGACTATT
ATTCAAATGACACAGAATGAAGACCTCCGAGAAAACATGT**TACGGCAAGTTCTGGAGCAT**TTGTCTCAGCAAAGT
GAAAGCCAGTACCTAAAGATTCTAACAAGCCTTGCTGAAGTTGCTACAACAAATGGTCATAAACTGCTTAGCCTCT
CTAGCAATTATGATGCTCAAATGAAGAGCCTTTTAAGGATTGTGAGAATGTTTTGTCACGTCTTTCGAATTGGTCCA
TCCTCCCCCAGTAATGGAATTGATATGGGCTACAATGGGAATAAAACTCCAAGAAGCCAGGTGTTCAAGCCTCTG
GAATTGCTTTGGCACTCGTTAGATGAATGGCTAGTTTTAATAGCCACAGAATTGATGAAAAACAAAAGAGACTCAA
CAGAGATCACTTCTATTTTACTGAAACAAAAAGGCCAAGATCAAGATGCTGCTTCCATTCCTCCATTTGAACCTCCA
GGACCTGGGAGCTATGAAAATCTGTCCACTGGCACAAGGGAATCTAAACCAGATGCTCTTGCAGGGAGACAGGA

AGCCAGTGCAGATTGTCAGGATGTTATTTCTATGACAGCTAACCGGCTAAGTGCTGTCATTCAAGCTTTTTACATGT
GCTGTTCTTGTCAGATGCCTCCGGGAATGACTTCACCTCGTTTCATTGAATTTGTCTGCAAACATGATGAAGTTTTA
AAATGCTTTGTTAATAGAAATCCCAAAATTATATTTGACCACTTTCACTTTCTCCTTGAATGTCCTGAGTTGATGTCA
AGATTCATGCATATCATAAAAGCACAGCCTTTTAAAGATCGCTGTGAATGGTTCTATGAACATTTGCATTCAGGAC
AGCCAGATTCAGATATGGTGCACAGGCCAGTGAATGAAAATGATATCCTGCTGGTTCACAGAGATTCTATTTTTAG
GAGTAGCTGTGAAGTTGTGTCAAAAGCAAATTGTGCAAAGCTAAAGCAAGGGATTGCTGTACGGTTCCATGGAGA
AGAAGGCATGGGTCAAGGTGTTGTGCGTGAGTGGTTTGATATTCTGTCCAATGAGATAGTCAATCCTGATTATGC
ATTGTTTACCCAGTCAGCTGATGGAACAACTTTTCAGCCTAATAGCAACTCTTATGTAAATCCTGATCACTTGAACT
ATTTTCGGTTTGCTGGGCAGATCTTGGGATTAGCGTTGAACCACAGGCAGCTGGTCAATATTTACTTCACACGATC
CTTCTACAAGCACATTCTTGGTATTCCTGTAAATTACCAAGATGTGGCATCCATTGATCCAGAATATGCGAAAAATT
TGCAATGGATTTTAGATAATGATATAAGTGATCTGGGTCTAGAACTAACTTTTTCTGTTGAGACTGATGTGTTTGGA
GCAATGGAAGAGGTGCCTTTGAAACCTGGGGGTGGGAGTATTCTTGTGACACAAAATAATAAAGCGGAGTACGT
CCAGCTTGTTACTGAACTTCGAATGACAAGAGCCATTCAGCCTCAGATCAATGCTTTTTTACAGGGCTTTCATATGT
TCATTCCACCCTCCCTCATACAGCTTTTTGATGAATATGAATTGGAGCTACTGCTTTCTGGCATGCCAGAAATTGAT
GTGAGTGATTGGATAAAAAATACAGAATACACAAGTGGCTATGAAAGAGAAGATCCAGTTATTCAGTGGTTCTGG
GAAGTTGTAGAAGACATTACTCAAGAGGAGAGAGTTCTTCTCTTACAGTTTGTTACGGGCAGTTCCAGGGTCCCAC
ATGGTGGGTTTGCTAATATCATGGGTGGAAGTGGATTGCAAAACTTTACAATCGCTGCTGTGCCATATACTCCAAA
TCTTTTACCAACTTCAAGCACATGCATCAACATGCTCAAGTTACCTGAATACCCAAGTAAAGAAATACTCAAGGACA
GACTTCTTGTGGCACTACATTGTGGCAGCTATGGTTACACAATGGCATAATGAAGTCTGGAAAACTCCTCTGACTA
CTGATGCACAATTCAGAATGGCAGAAGTAATTTGGGAAAATGTCAACAAAAAAGCAGCCTAAATGCAACCCATAG
GCAGGGCTGATGCTTCCAATTTATAAAGGATCATCAGGTTTTCTGTTTCTCTCTTTTCCCTTTTATGTTTTCTCTGTTT
GTGATACAATTAGAAAATATAAAATCACAGTAGATTTTATTTTTTAAAATGCTAACTGAAAGTAATAGAGACTGTCC
TTTTTCATAATTAATTTTATCCAAGATTGTATTAAGGCAAAATCTGATTCTACATTCCACCTCTGCTATGTAACTGTCT
TGTTAAAAGGGTGTTTTCTCCTAATTTCTGATATATTATATGAGGTCATCCAGCTGGTGTGTTCTTTTGCATGTAAAC
TGCCATTTATATTTTAGAAAACTATTGTATAGAATGGATTTAGATTGTCTATAAAGCCACAAATACGTATTTTGCCA
CAGTGTATTCTATATTGCAATGATTTTTTTAGCATTTTAATATTTTAATATATATTGTAAAATTTAGACTGATGATACT
AACAGTTGATGAAATGACATATAATTTATATATGAAAGCTTACGCTATATTGTATGAATTATTTGCATCTTTCAGTG
GCCAGTTTTCCATATGTATATATTATGGTCTCAATGTTTTTCTTACGCCTCATTTTAATTTATAATGAAGGTAAAATT
AAAATGTATTTTACCACGTTTCTTTTCATTACTTTTATCTGTGAGCTCTGACACATCTGAAAAAGTAATCTGATGTGC
AAATTATAATTTAAATATGTTAATTTTTTTGCTTCTTAAATTTGCTTTTCATCATTAAAATGTCAAGTTCAAGTGATAT
GTGCCTAATATCACTTGGATGTTGGTGGGTTTTTGAATTTTTGGGTGGTTAATCAGTTTTATTTTGAAAAGACGTAC
TTGAATAGTTACAGCATATGTTTGAACAGGAAGTAGGAACATGCATACACGAAGAAATGCTAACGGAAGGATTTG
TTATGTTTAGGATCTTCCCTTGGAAACTAAAAATAGAATATTAATGACATTACTGTTTGTAGAATGACATATGCAGA
TTTTCTCATAAGCAGTCATTGTGTTTGCCAGTAATGTTTGAGAGACATGTAAGTTGAAAGTTTTGCTAAATTATAAA
GCTCCTTTAATTCGTTGGTTTTGATTCTCTTATTCTCTTGTCTTTTCTAAATGTTAACAAAATATATCTTAACAGATTA
CATGAAATTTAGGAATTATTTAAAAGTTACCATTAGCTCTAAAATTAAGATTCGGATGCTTTATTTATAGTAACTGA
AGCTAATAATGTTTTATGTTTTGATTTTTTGAAATTTAATTGTAGAAGTCACTGCCTTCTGAGTTTTCAAATAGATAA
CCACCTTTAATATTACACTGCTTATAATACTAATGTTTACAGATATGTTTCTGTTTATAACCATATAATACATTGGCTT
TGTCATATTAGTTTTTTTTGCAAGTAGTTATGTAAAAGAGATAGATAATAAAATATTAAATAACTGA

Isoform e (831 aa) = transcript variant 6 (4501 bp)

AGAAGGCCACGCGAGCCCGGGAGGGACGCGGCGGCGGGGGCTGCAGGAAAGGCGCGAGCAGAGGCGGCGGC
GGGTGTACTGTAGGTGGTCGGTCCGGCAGCAGCCCGGCCCCCGGACGCAGGACGTGGCCCCAGGCAGCCCTCGC
AGCTCAGTGCTCTAGCCGGGGCAAGCCCGCGTCTCCGCCTGCTGGACGGGCCCAGGCGAGATGTAGGGCTCTGG
GCGCGGAGGCCGCCGGTGGGGCGGCTGATCGCGGAGGATCGCGGAGGGCGCGCCGAGGATGGAGAGAGCGAT
GGAGCAACTCAACCGCCTGACGCGCTCGCTGCGCCGCGCGCACCGTGGAGTTGCCCGAGGAGCTGAGAGGCG
GCCCCGGGCCCCGAACTCCCCTGGCCCGACGCTCCCACCCCGGCAGATAATGAAACTGCTGTTTATACATTAATGC
CAATGGTTATGGCTGATCAACACAGGTCTGTTTCTGAACTACTATCAAATTCAAAATTTGATGTCAATTATGCATTC
GGACGTGTGAAAAGAAGCTTGCTTCACATTGCAGCAAATTGTGGATCGGTGGAATGCTTGGTTTTGCTGTTAAAG
AAAGGAGCAAATCCTAACTATCAAGATATTTCAGGCTGTACACCCCTTCA**TTTGGCAGCAAGAAATGGGC**AGAAG

AAATGTATGAGTAAATTATTAGAATATAGCGCTGATGTCAACATTTGTAATAATGAAGGCCTTACAGCA**ACAGTGC**
AGTGCTTGCTAGACAGTGGTGCTGATATTAACAGGCCAAATGTATCAGGAGCAACTCCATTGTACTTTGCTTGCA
GTCATGGTCAGAGAGATACAGCACAGATCCTACTATTACGAGGAGCCAAATATCTGCCAGATAAAAATGGAGTAA
CTCCTCTGGATTTATGTGTACAGGGTGGATATGGAGAGACTTGTGAAGTATTAATTCAATATCACCCGAGGCTTTT
TCAGACTATTATTCAAATGACACAGAATGAAGACCTCCGAGAAAACATGT**TACGGCAAGTTCTGGAGCAT**TTGTCT
CAGCAAAGTGAAAGCCAGTACCTAAAGATTCTAACAAGCCTTGCTGAAGTTGCTACAACAAATGGTCATAAACTGC
TTAGCCTCTCTAGCAATTATGATGCTCAAATGAAGAGCCTTTTAAGGATTGTGAGAATGTTTTGTCACGTCTTTCGA
ATTGGTCCATCCTCCCCCAGTAATGGAATTGATATGGGCTACAATGGGAATAAAACTCCAAGAAGCCAGGTGTTCA
AGCCTCTGGAATTGCTTTGGCACTCGTTAGATGAATGGCTAGTTTTAATAGCCACAGAATTGATGAAAAACAAAAG
AGACTCAACAGAGATCACTTCTATTTTACTGAAACAAAAAGGCCAAGATCAAGATGCTGCTTCCATTCCTCCATTTG
AACCTCCAGGACCTGGGAGCTATGAAAATCTGTCCACTGGCACAAGGGAATCTAAACCAGATGCTCTTGCAGGGA
GACAGGAAGCCAGTGCAGATTGTCAGGATGTTATTTCTATGACAGCTAACCGGCTAAGTGCTGTCATTCAAGCTTT
TTACATGTGCTGTTCTTGTCAGATGCCTCCGGGAATGACTTCACCTCGTTTCATTGAATTTGTCTGCAAACATGATG
AAGTTTTAAAATGCTTTGTTAATAGAAATCCCAAAATTATATTTGACCACTTTCACTTTCTCCTTGAATGTCCTGAGT
TGATGTCAAGATTCATGCATATCATAAAAGCACAGCCTTTTAAAGATCGCTGTGAATGGTTCTATGAACATTTGCAT
TCAGGACAGCCAGATTCAGATATGGTGCACAGGCCAGTGAATGAAAATGATATCCTGCTGGTTCACAGAGATTCT
ATTTTTAGGAGTAGCTGTGAAGTTGTGTCAAAAGCAAATTGTGCAAAGCTAAAGCAAGGGATTGCTGTACGGTTC
CATGGAGAAGAAGGCATGGGTCAAGGTGTTGTGCGTGAGTGGTTTGATATTCTGTCCAATGAGATAGTCAATCCT
GATTATGCATTGTTTACCCAGTCAGCTGATGGAACAACTTTTCAGCCTAATAGCAACTCTTATGTAAATCCTGATCA
CTTGAACTATTTTCGGTTTGCTGGGCAGATCTTGGGATTAGCGTTGAACCACAGGCAGCTGGTCAATATTTACTTCA
CACGATCCTTCTACAAGCACATTCTTGGTATTCCTGTAAATTACCAAGATGTGGCATCCATTGATCCAGAATATGCG
AAAAATTTGCAATGGATTTTAGATAATGATATAAGTGATCTGGGTCTAGAACTAACTTTTTCTGTTGAGACTGATGT
GTTTGGAGCAATGGAAGAGGTGCCTTTGAAACCTGGGGGTGGGAGTATTCTTGTGACACAAAATAATAAAGCGG
AGTACGTCCAGCTTGTTACTGAACTTCGAATGACAAGAGCCATTCAGCCTCAGATCAATGCTTTTTTACAGGGCTTT
CATATGTTCATTCCACCCTCCCTCATACAGCTTTTTGATGAATATGAATTGGAGCTACTGCTTTCTGGCATGCCAGA
AATTGATGTGAGTGATTGGATAAAAAATACAGAATACACAAGTGGCTATGAAAGAGAAGATCCAGTTATTCAGTG
GTTCTGGGAAGTTGTAGAAGACATTACTCAAGAGGAGAGAGTTCTTCTCTTACAGTTTGTTACGGGCAGTTCCAGG
GTCCCACATGGTGGGTTTGCTAATATCATGGGTGGAAGTGGATTGCAAAACTTTACAATCGCTGCTGTGCCATATA
CTCCAAATCTTTTACCAACTTCAAGCACATGCATCAACATGCTCAAGTTACCTGAATACCCAAGTAAAGAAATACTC
AAGGACAGACTTCTTGTGGCACTACATTGTGGCAGCTATGGTTACACAATGGCATAATGAAGTCTGGAAAACTCCT
CTGACTACTGATGCACAATTCAGAATGGCAGAAGTAATTTGGGAAAATGTCAACAAAAAAGCAGCCTAAATGCAA
CCCATAGGCAGGGCTGATGCTTCCAATTTATAAAGGATCATCAGGTTTTCTGTTTCTCTCTTTTCCCTTTTATGTTTTC
TCTGTTTGTGATACAATTAGAAAATATAAAATCACAGTAGATTTTATTTTTTAAAATGCTAACTGAAAGTAATAGAG
ACTGTCCTTTTTCATAATTAATTTTATCCAAGATTGTATTAAGGCAAAATCTGATTCTACATTCCACCTCTGCTATGTA
ACTGTCTTGTTAAAAGGGTGTTTTCTCCTAATTTCTGATATATTATATGAGGTCATCCAGCTGGTGTGTTCTTTTGCA
TGTAAACTGCCATTTATATTTTAGAAAACTATTGTATAGAATGGATTTAGATTGTCTATAAAGCCACAAATACGTAT
TTTGCCACAGTGTATTCTATATTGCAATGATTTTTTTAGCATTTTAATATTTTAATATATATTGTAAAATTTAGACTGA
TGATACTAACAGTTGATGAAATGACATATAATTTATATATGAAAGCTTACGCTATATTGTATGAATTATTTGCATCTT
TCAGTGGCCAGTTTTCCATATGTATATATTATGGTCTCAATGTTTTTCTTACGCCTCATTTTAATTTATAATGAAGGT
AAAATTAAAATGTATTTTACCACGTTTCTTTTCATTACTTTTATCTGTGAGCTCTGACACATCTGAAAAAGTAATCTG
ATGTGCAAATTATAATTTAAATATGTTAATTTTTTTGCTTCTTAAATTTGCTTTTCATCATTAAAATGTCAAGTTCAAG
TGATATGTGCCTAATATCACTTGGATGTTGGTGGGTTTTTGAATTTTTGGGTGGTTAATCAGTTTTATTTTGAAAAG
ACGTACTTGAATAGTTACAGCATATGTTTGAACAGGAAGTAGGAACATGCATACACGAAGAAATGCTAACGGAAG
GATTTGTTATGTTTAGGATCTTCCCTTGGAAACTAAAAATAGAATATTAATGACATTACTGTTTGTAGAATGACATA
TGCAGATTTTCTCATAAGCAGTCATTGTGTTTGCCAGTAATGTTTGAGAGACATGTAAGTTGAAAGTTTTGCTAAAT
TATAAAGCTCCTTTAATTCGTTGGTTTTGATTCTCTTATTCTCTTGTCTTTTCTAAATGTTAACAAAATATATCTTAAC
AGATTACATGAAATTTAGGAATTATTTAAAAGTTACCATTAGCTCTAAAATTAAGATTCGGATGCTTTATTTATAGT
AACTGAAGCTAATAATGTTTTATGTTTTGATTTTTTGAAATTTAATTGTAGAAGTCACTGCCTTCTGAGTTTTCAAAT
AGATAACCACCTTTAATATTACACTGCTTATAATACTAATGTTTACAGATATGTTTCTGTTTATAACCATATAATACA
TTGGCTTTGTCATATTAGTTTTTTTTGCAAGTAGTTATGTAAAAGAGATAGATAATAAAATATTAAATAACTGA

Isoform b (865 aa) = Transcript variant 3 (4443 bp)

AGAAGGCCACGCGAGCCCGGGAGGGACGCGGCGGCGGGGGCTGCAGGAAAGGCGCGAGCAGAGGCGGCGGC
GGGTGTACTGTAGGTGGTCGGTCCGGCAGCAGCCCGGCCCCCGGACGCAGGACGTGGCCCCAGGCAGCCCTCGC
AGCTCAGTGCTCTAGCCGGGGCAAGCCCGCGTCTCCGCCTGCTGGACGGGCCCAGGCGAGATGTAGGGCTCTGG
GCGCGGAGGCCGCCGGTGGGGCGGCTGATCGCGGAGGATCGCGGAGGGCGCGCCGAGGATGGAGAGAGCGAT
GGAGCAACTCAACCGCCTGACGCGCTCGCTGCGCCGCGCGCGCACCGTGGAGTTGCCCGAGGATAATGAAACTG
CTGTTTATACATTAATGCCAATGGTTATGGCTGATCAACACAGGTCTGTTTCTGAACTACTATCAAATTCAAAATTT
GATGTCAATTATGCATTCGGACGTGTGAAAAGAAGCTTGCTTCACATTGCAGCAAATTGTGGATCGGTGGAATGCT
TGGTTTTGCTGTTAAAGAAAGGAGCAAATCCTAACTATCAAGATATTTCAGGCTGTACACCCCTTCA**TTTGGCAGC**
AAGAAATGGGCAGAAGAAATGTATGAGTAAATTATTAGAATATAGCGCTGATGTCAACATTTGTAATAATGAAGG
CCTTACAGCA**ACAGTGCAGTGCTTGCTAGACAGTGGTGCTGATATTAACAGGCCAAATGTATCAGGAGCAACTC**
CATTGTACTTTGCTTGCAGTCATGGTCAGAGAGATACAGCACAGATCCTACTATTACGAGGAGCCAAATATCTGCC
AGATAAAAATGGAGTAACTCCTCTGGATTTATGTGTACAGGGTGGATATGGAGAGACTTGTGAAGTATTAATTCA
ATATCACCCGAGGCTTTTTCAGACTATTATTCAAATGACACAGAATGAAGACCTCCGAGAAAACATGT**TACGGCAA**
GTTCTGGAGCATTTGTCTCAGCAAAGTGAAAGCCAGTACCTAAAGATTCTAACAAGCCTTGCTGAAGTTGCTACAA
CAAATGGTCATAAACTGCTTAGCCTCTCTAGCAATTATGATGCTCAAATGAAGAGCCTTTTAAGGATTGTGAGAAT
GTTTTGTCACGTCTTTCGAATTGGTCCATCCTCCCCCAGTAATGGAATTGATATGGGCTACAATGGGAATAAAACTC
CAAGAAGCCAGGTGTTCAAGCCTCTGGAATTGCTTTGGCACTCGTTAGATGAATGGCTAGTTTTAATAGCCACAGA
ATTGATGAAAAACAAAAGAGACTCAACAGAGATCACTTCTATTTTACTGAAACAAAAAGGCCAAGATCAAGATGC
TGCTTCCATTCCTCCATTTGAACCTCCAGGACCTGGGAGCTATGAAAATCTGTCCACTGGCACAAGGGAATCTAAA
CCAGATGCTCTTGCAGGGAGACAGGAAGCCAGTGCAGATTGTCAGGATGTTATTTCTATGACAGCTAACCGGCTA
AGTGCTGTCATTCAAGCTTTTTACATGTGCTGTTCTTGTCAGATGCCTCCGGGAATGACTTCACCTCGTTTCATTGAA
TTTGTCTGCAAACATGATGAAGTTTTAAAATGCTTTGTTAATAGAAATCCCAAAATTATATTTGACCACTTTCACTTT
CTCCTTGAATGTCCTGAGTTGATGTCAAGATTCATGCATATCATAAAAGCACAGCCTTTTAAAGATCGCTGTGAATG
GTTCTATGAACATTTGCATTCAGGACAGCCAGATTCAGATATGGTGCACAGGCCAGTGAATGAAAATGATATCCTG
CTGGTTCACAGAGATTCTATTTTTAGGAGTAGCTGTGAAGTTGTGTCAAAAGCAAATTGTGCAAAGCTAAAGCAAG
GGATTGCTGTACGGTTCCATGGAGAAGAAGGCATGGGTCAAGGTGTTGTGCGTGAGTGGTTTGATATTCTGTCCA
ATGAGATAGTCAATCCTGATTATGCATTGTTTACCCAGTCAGCTGATGGAACAACTTTTCAGCCTAATAGCAACTCT
TATGTAAATCCTGATCACTTGAACTATTTTCGGTTTGCTGGGCAGATCTTGGGATTAGCGTTGAACCACAGGCAGC
TGGTCAATATTTACTTCACACGATCCTTCTACAAGCACATTCTTGGTATTCCTGTAAATTACCAAGATGTGGCATCCA
TTGATCCAGAATATGCGAAAAATTTGCAATGGATTTTAGATAATGATATAAGTGATCTGGGTCTAGAACTAACTTTT
TCTGTTGAGACTGATGTGTTTGGAGCAATGGAAGAGGTGCCTTTGAAACCTGGGGGTGGGAGTATTCTTGTGACA
CAAAATAATAAAGCGGAGTACGTCCAGCTTGTTACTGAACTTCGAATGACAAGAGCCATTCAGCCTCAGATCAATG
CTTTTTTACAGGGCTTTCATATGTTCATTCCACCCTCCCTCATACAGCTTTTTGATGAATATGAATTGGAGCTACTGC
TTTCTGGCATGCCAGAAATTGATGTGAGTGATTGGATAAAAAATACAGAATACACAAGTGGCTATGAAAGAGAAG
ATCCAGTTATTCAGTGGTTCTGGGAAGTTGTAGAAGACATTACTCAAGAGGAGAGAGTTCTTCTCTTACAGTTTGT
TACGGGCAGTTCCAGGGTCCCACATGGTGGGTTTGCTAATATCATGGGTGGAAGTGGATTGCAAAACTTTACAAT
CGCTGCTGTGCCATATACTCCAAATCTTTTACCAACTTCAAGCACATGCATCAACATGCTCAAGTTACCTGAATACC
CAAGTAAAGAAATACTCAAGGACAGACTTCTTGTGGCACTACATTGTGGCAGCTATGGTTACACAATGGCATAATG
AAGTCTGGAAAACTCCTCTGACTACTGATGCACAATTCAGAATGGCAGAAGTAATTTGGGAAAATGTCAACAAAA
AAGCAGCCTAAATGCAACCCATAGGCAGGGCTGATGCTTCCAATTTATAAAGGATCATCAGGTTTTCTGTTTCTCTC
TTTTCCCTTTTATGTTTTCTCTGTTTGTGATACAATTAGAAAATATAAAATCACAGTAGATTTTATTTTTTAAAATGCT
AACTGAAAGTAATAGAGACTGTCCTTTTTCATAATTAATTTTATCCAAGATTGTATTAAGGCAAAATCTGATTCTAC
ATTCCACCTCTGCTATGTAACTGTCTTGTTAAAAGGGTGTTTTCTCCTAATTTCTGATATATTATATGAGGTCATCCA
GCTGGTGTGTTCTTTTGCATGTAAACTGCCATTTATATTTTAGAAAACTATTGTATAGAATGGATTTAGATTGTCTAT
AAAGCCACAAATACGTATTTTGCCACAGTGTATTCTATATTGCAATGATTTTTTTAGCATTTTAATATTTTAATATAT
ATTGTAAAATTTAGACTGATGATACTAACAGTTGATGAAATGACATATAATTTATATATGAAAGCTTACGCTATATT
GTATGAATTATTTGCATCTTTCAGTGGCCAGTTTTCCATATGTATATATTATGGTCTCAATGTTTTTCTTACGCCTCAT
TTTAATTTATAATGAAGGTAAAATTAAAATGTATTTTACCACGTTTCTTTTCATTACTTTTATCTGTGAGCTCTGACAC

ATCTGAAAAAGTAATCTGATGTGCAAATTATAATTTAAATATGTTAATTTTTTTGCTTCTTAAATTTGCTTTTCATCAT
TAAAATGTCAAGTTCAAGTGATATGTGCCTAATATCACTTGGATGTTGGTGGGTTTTTGAATTTTTGGGTGGTTAAT
CAGTTTTATTTTGAAAAGACGTACTTGAATAGTTACAGCATATGTTTGAACAGGAAGTAGGAACATGCATACACGA
AGAAATGCTAACGGAAGGATTTGTTATGTTTAGGATCTTCCCTTGGAAACTAAAAATAGAATATTAATGACATTAC
TGTTTGTAGAATGACATATGCAGATTTTCTCATAAGCAGTCATTGTGTTTGCCAGTAATGTTTGAGAGACATGTAA
GTTGAAAGTTTTGCTAAATTATAAAGCTCCTTTAATTCGTTGGTTTTGATTCTCTTATTCTCTTGTCTTTTCTAAATGT
TAACAAAATATATCTTAACAGATTACATGAAATTTAGGAATTATTTAAAAGTTACCATTAGCTCTAAAATTAAGATT
CGGATGCTTTATTTATAGTAACTGAAGCTAATAATGTTTTATGTTTTGATTTTTTGAAATTTAATTGTAGAAGTCACT
GCCTTCTGAGTTTTCAAATAGATAACCACCTTTAATATTACACTGCTTATAATACTAATGTTTACAGATATGTTTCTG
TTTATAACCATATAATACATTGGCTTTGTCATATTAGTTTTTTTGCAAGTAGTTATGTAAAAGAGATAGATAATAAA
ATATTAAATAACTGA

Isoform h (715 aa) = Transcript variant 11 (4360 bp)

AGAAGGCCACGCGAGCCCGGGAGGGACGCGGCGGCGGGGGCTGCAGGAAAGGCGCGAGCAGAGGCGGCGGC
GGGTGTACTGTAGGTGGTCGGTCCGGCAGCAGCCCGGCCCCCGGACGCAGGACGTGGCCCCAGGCAGCCCTCGC
AGCTCAGTGCTCTAGCCGGGGCAAGCCCGCGTCTCCGCCTGCTGGACGGGCCCAGGCGAGATGTAGGGCTCTGG
GCGCGGAGGCCGCCGGTGGGGCGGCTGATCGCGGAGGATCGCGGAGGGCGCGCCGAGGATGGAGAGAGCGAT
GGAGCAACTCAACCGCCTGACGCGCTCGCTGCGCCGCGCGCGCACCGTGGAGTTGCCCGAGGATAATGAAACTG
CTGTTTATACATTAATGCCAATGGTTATGGCTGATCAACACAGGTCTGTTTCTGAACTACTATCAAATTCAAAATTT
GATGTCAATTATGCATTCGGACGTGTGAAAAGAAGCTTGCTTCACATTGCAGCAAATTGTGGATCGGTGGAATGCT
TGGTTTTGCTGTTAAAGAAAGGAGCAAATCCTAACTATCAAGATATTTCAGGCTGTACACCCCTTCA**TTTGGCAGC**
AAGAAATGGGCAGAAGAAATGTATGAGTAAATTATTAGAATATAGCGCTGATGTCAACATTTGTAATAATGAAGG
CCTTACAGCATCATGGTCAGAGAGATACAGCACAGATCCTACTATTACGAGGAGCCAAATATCTGCCAGATAAAAA
TGGAGTAACTCCTCTGGATTTATGTGTACAGGGTGGATATGGAGAGACTTGTGAAGTATTAATTCAATATCACCCG
AGGCTTTTTCAGACTATTATTCAAATGACACAGAATGAAGACCTCCGAGAAAACATGT**TACGGCAAGTTCTGGAG**
CATTTGTCTCAGCAAAGTGAAAGCCAGTACCTAAAGATTCTAACAAGCCTTGCTGAAGTTGCTACAACAAATGGTC
ATAAACTGCTTAGCCTCTCTAGCAATTATGATGCTCAAATGAAGAGCCTTTTAAGGATTGTGAGAATGTTTTGTCAC
GTCTTTCGAATTGGTCCATCCTCCCCCAGTAATGGAATTGATATGGGCTACAATGGGAATAAAACTCCAAGAAGCC
AGGTGTTCAAGCCTCTGGAATTGCTTTGGCACTCGTTAGATGAATGGCTAGTTTTAATAGCCACAGAATTGATGAA
AAACAAAAGAGACTCAACAGAGATCACTTCTATTTTACTGAAACAAAAAGGCCAAGATCAAGATGCTGCTTCCATT
CCTCCATTTGAACCTCCAGGACCTGGGAGCTATGAAAATCTGTCCACTGGCACAAGGGAATCTAAACCAGATGCTC
TTGCAGGGAGACAGGAAGCCAGTGCAGATTGTCAGGATGTTATTTCTATGACAGCTAACCGGCTAAGTGCTGTCA
TTCAAGCTTTTTACATGTGCTGTTCTTGTCAGATGCCTCCGGGAATGACTTCACCTCGTTTCATTGAATTTGTCTGCA
AACATGATGAAGTTTTAAAATGCTTTGTTAATAGAAATCCCAAAATTATATTTGACCACTTTCACTTTCTCCTTGAAT
GTCCTGAGTTGATGTCAAGATTCATGCATATCATAAAAGCACAGCCTTTTAAAGATCGCTGTGAATGGTTCTATGA
ACATTTGCATTCAGGACAGCCAGATTCAGATATGGTGCACAGGCCAGTGAATGAAAATGATATCCTGCTGGTTCAC
AGAGATTCTATTTTTAGGAGTAGCTGTGAAGTTGTGTCAAAAGCAAATTGTGCAAAGCTAAAGCAAGGGATTGCT
GTACGGTTCCATGGAGAAGAAGGCATGGGTCAAGGTGTTGTGCGTGAGTGGTTTGATATTCTGTCCAATGAGATA
GTCAATCCTGATTATGCATTGTTTACCCAGTCAGCTGATGGAACAACTTTTCAGCCTAATAGCAACTCTTATGTAAA
TCCTGATCACTTGAACTATTTTCGGTTTGCTGGGCAGATCTTGGGATTAGCGTTGAACCACAGGCAGCTGGTCAAT
ATTTACTTCACACGATCCTTCTACAAGCACATTCTTGGTATTCCTGTAAATTACCAAGATGTGGCATCCATTGATCCA
GAATATGCGAAAAATTTGCAATGGATTTTAGATAATGATATAAGTGATCTGGGTCTAGAACTAACTTTTTCTGTTGA
GACTGATGTGTTTGGAGCAATGGAAGAGGTGCCTTTGAAACCTGGGGGTGGGAGTATTCTTGTGACACAAAATAA
TAAAGCGGAGTACGTCCAGCTTGTTACTGAACTTCGAATGACAAGAGCCATTCAGCCTCAGATCAATGCTTTTTTAC
AGGGCTTTCATATGTTCATTCCACCCTCCCTCATACAGCTTTTTGATGAATATGAATTGGAGCTACTGCTTTCTGGCA
TGCCAGAAATTGATGTGAGTGATTGGATAAAAAATACAGAATACACAAGTGGCTATGAAAGAGAAGATCCAGTTA
TTCAGTGGTTCTGGGAAGTTGTAGAAGACATTACTCAAGAGGAGAGAGTTCTTCTCTTACAGTTTGTTACGGGCAG
TTCCAGGGTCCCACATGGTGGGTTTGCTAATATCATGGGTGGAAGTGGATTGCAAAACTTTACAATCGCTGCTGTG
CCATATACTCCAAATCTTTTACCAACTTCAAGCACATGCATCAACATGCTCAAGTTACCTGAATACCCAAGTAAAGA
AATACTCAAGGACAGACTTCTTGTGGCACTACATTGTGGCAGCTATGGTTACACAATGGCATAATGAAGTCTGGAA

AACTCCTCTGACTACTGATGCACAATTCAGAATGGCAGAAGTAATTTGGGAAAATGTCAACAAAAAAGCAGCCTA
AATGCAACCCATAGGCAGGGCTGATGCTTCCAATTTATAAAGGATCATCAGGTTTTCTGTTTCTCTCTTTTCCCTTTT
ATGTTTTCTCTGTTTGTGATACAATTAGAAAATATAAAATCACAGTAGATTTTATTTTTTAAAATGCTAACTGAAAGT
AATAGAGACTGTCCTTTTTCATAATTAATTTTATCCAAGATTGTATTAAGGCAAAATCTGATTCTACATTCCACCTCT
GCTATGTAACTGTCTTGTTAAAAGGGTGTTTTCTCCTAATTTCTGATATATTATATGAGGTCATCCAGCTGGTGTGTT
CTTTTGCATGTAAACTGCCATTTATATTTTAGAAAACTATTGTATAGAATGGATTTAGATTGTCTATAAAGCCACAA
ATACGTATTTTGCCACAGTGTATTCTATATTGCAATGATTTTTTTAGCATTTTAATATTTTAATATATATTGTAAAATT
TAGACTGATGATACTAACAGTTGATGAAATGACATATAATTTATATATGAAAGCTTACGCTATATTGTATGAATTAT
TTGCATCTTTCAGTGGCCAGTTTTCCATATGTATATATTATGGTCTCAATGTTTTTCTTACGCCTCATTTTAATTTATA
ATGAAGGTAAAATTAAAATGTATTTTACCACGTTTCTTTTCATTACTTTTATCTGTGAGCTCTGACACATCTGAAAAA
GTAATCTGATGTGCAAATTATAATTTAAATATGTTAATTTTTTTGCTTCTTAAATTTGCTTTTCATCATTAAAATGTCA
AGTTCAAGTGATATGTGCCTAATATCACTTGGATGTTGGTGGGTTTTTGAATTTTTGGGTGGTTAATCAGTTTTATT
TTGAAAAGACGTACTTGAATAGTTACAGCATATGTTTGAACAGGAAGTAGGAACATGCATACACGAAGAAATGCT
AACGGAAGGATTTGTTATGTTTAGGATCTTCCCTTGGAAACTAAAAATAGAATATTAATGACATTACTGTTTGTAGA
ATGACATATGCAGATTTTCTCATAAGCAGTCATTGTGTTTGCCAGTAATGTTTGAGAGACATGTAAGTTGAAAGTTT
TGCTAAATTATAAAGCTCCTTTAATTCGTTGGTTTTGATTCTCTTATTCTCTTGTCTTTTCTAAATGTTAACAAAATAT
ATCTTAACAGATTACATGAAATTTAGGAATTATTTAAAAGTTACCATTAGCTCTAAAATTAAGATTCGGATGCTTTA
TTTATAGTAACTGAAGCTAATAATGTTTTATGTTTTGATTTTTTGAAATTTAATTGTAGAAGTCACTGCCTTCTGAGT
TTTCAAATAGATAACCACCTTTAATATTACACTGCTTATAATACTAATGTTTACAGATATGTTTCTGTTTATAACCATA
TAATACATTGGCTTTGTCATATTAGTTTTTTTTGCAAGTAGTTATGTAAAAGAGATAGATAATAAAATATTAAATAA
CTGA

Protein sequences

Isoform a (909 aa)

MERAMEQLNRLTRSLRRARTVELPEDNETAVYTLMPMVMADQHRSVSELLSNSKFDVNYAFGRVKRSLLHIAANCGS
VECLVLLLKKGANPNYQDISGCTPLHLAARNGQKKCMSKLLEYSADVNICNNEGLTAIHWLAVNGRTELLHDLVQHVS
DVDVEDAMGQTALHVACQNGHKTTVQCLLDSGADINRPNVSGATPLYFACSHGQRDTAQILLLRGAKYLPDKNGVTP
LDLCVQGGYGETCEVLIQYHPRLFQTIIQMTQNEDLRENMLRQVLEHLSQQSESQYLKILTSLAEVATTNGHKLLSLSSNY
DAQMKSLLRIVRMFCHVFRIGPSSPSNGIDMGYNGNKTPRSQVFKPLELLWHSLDEWLVLIATELMKNKRDSTEITSILL
KQKGQDQDAASIPPFEPPGPGSYENLSTGTRESKPDALAGRQEASADCQDVISMTANRLSAVIQAFYMCCSCQMPPG
MTSPRFIEFVCKHDEVLKCFVNRNPKIIFDHFHFLLECPELMSRFMHIIKAQPFKDRCEWFYEHLHSGQPDSDMVHRPV
NENDILLVHRDSIFRSSCEVVSKANCAKLKQGIAVRFHGEEGMGQGVVREWFDILSNEIVNPDYALFTQSADGTTFQPN
SNSYVNPDHLNYFRFAGQILGLALNHRQLVNIYFTRSFYKHILGIPVNYQDVASIDPEYAKNLQWILDNDISDLGLELTFSV
ETDVFGAMEEVPLKPGGGSILVTQNNKAEYVQLVTELRMTRAIQPQINAFLQGFHMFIPPSLIQLFDEYELELLLSGMPE
IDVSDWIKNTEYTSGYEREDPVIQWFWEVVEDITQEERVLLLQFVTGSSRVPHGGFANIMGGSGLQNFTIAAVPYTPNL
LPTSSTCINMLKLPEYPSKEILKDRLLVALHCGSYGYTMA

HACE1 protein as long as exon 7 is deleted

MERAMEQLNRLTRSLRRARTVELPEDNETAVYTLMPMVMADQHRSVSELLSNSKFDVNYAFGRVKRSLLHIAANCGS
VECLVLLLKKGANPNYQDISGCTPLHLAARNGQKKCMSKLLEYSADVNICNNEGLTAIHWLAVNGRTELLHDLVQHVS
DVDVEDAMGQTALHVACQNGHKTSWSERYSTDPTITRSQISAR*

Isoform d (741 aa)

MGGQNYSMTLCSMSVMLMLRMPWGRQHCMLPARTVTRRHGQRDTAQILLLRGAKYLPDKNGVTPLDLCVQGGY
GETCEVLIQYHPRLFQTIIQMTQNEDLRENMLRQVLEHLSQQSESQYLKILTSLAEVATTNGHKLLSLSSNYDAQMKSLL
RIVRMFCHVFRIGPSSPSNGIDMGYNGNKTPRSQVFKPLELLWHSLDEWLVLIATELMKNKRDSTEITSILLKQKGQDQ
DAASIPPFEPPGPGSYENLSTGTRESKPDALAGRQEASADCQDVISMTANRLSAVIQAFYMCCSCQMPPGMTSPRFIEF
VCKHDEVLKCFVNRNPKIIFDHFHFLLECPELMSRFMHIIKAQPFKDRCEWFYEHLHSGQPDSDMVHRPVNENDILLVH
RDSIFRSSCEVVSKANCAKLKQGIAVRFHGEEGMGQGVVREWFDILSNEIVNPDYALFTQSADGTTFQPNSNSYVNPD
HLNYFRFAGQILGLALNHRQLVNIYFTRSFYKHILGIPVNYQDVASIDPEYAKNLQWILDNDISDLGLELTFSVETDVFGA
MEEVPLKPGGGSILVTQNNKAEYVQLVTELRMTRAIQPQINAFLQGFHMFIPPSLIQLFDEYELELLLSGMPEIDVSDWI
KNTEYTSGYEREDPVIQWFWEVVEDITQEERVLLLQFVTGSSRVPHGGFANIMGGSGLQNFTIAAVPYTPNLLPTSSTCI
NMLKLPEYPSKEILKDRLLVALHCGSYGYTMA

Isoform e (831 aa)

MPMVMADQHRSVSELLSNSKFDVNYAFGRVKRSLLHIAANCGSVECLVLLLKKGANPNYQDISGCTPLHLAARNGQKK
CMSKLLEYSADVNICNNEGLTATVQCLLDSGADINRPNVSGATPLYFACSHGQRDTAQILLLRGAKYLPDKNGVTPLDLC
VQGGYGETCEVLIQYHPRLFQTIIQMTQNEDLRENMLRQVLEHLSQQSESQYLKILTSLAEVATTNGHKLLSLSSNYDAQ
MKSLLRIVRMFCHVFRIGPSSPSNGIDMGYNGNKTPRSQVFKPLELLWHSLDEWLVLIATELMKNKRDSTEITSILLKQK
GQDQDAASIPPFEPPGPGSYENLSTGTRESKPDALAGRQEASADCQDVISMTANRLSAVIQAFYMCCSCQMPPGMTS
PRFIEFVCKHDEVLKCFVNRNPKIIFDHFHFLLECPELMSRFMHIIKAQPFKDRCEWFYEHLHSGQPDSDMVHRPVNEN
DILLVHRDSIFRSSCEVVSKANCAKLKQGIAVRFHGEEGMGQGVVREWFDILSNEIVNPDYALFTQSADGTTFQPNSNS
YVNPDHLNYFRFAGQILGLALNHRQLVNIYFTRSFYKHILGIPVNYQDVASIDPEYAKNLQWILDNDISDLGLELTFSVET
DVFGAMEEVPLKPGGGSILVTQNNKAEYVQLVTELRMTRAIQPQINAFLQGFHMFIPPSLIQLFDEYELELLLSGMPEID
VSDWIKNTEYTSGYEREDPVIQWFWEVVEDITQEERVLLLQFVTGSSRVPHGGFANIMGGSGLQNFTIAAVPYTPNLLP
TSSTCINMLKLPEYPSKEILKDRLLVALHCGSYGYTMA

Isoform b (865 aa)

MERAMEQLNRLTRSLRRARTVELPEDNETAVYTLMPMVMADQHRSVSELLSNSKFDVNYAFGRVKRSLLHIAANCGS
VECLVLLLKKGANPNYQDISGCTPLHLAARNGQKKCMSKLLEYSADVNICNNEGLTATVQCLLDSGADINRPNVSGATP
LYFACSHGQRDTAQILLLRGAKYLPDKNGVTPLDLCVQGGYGETCEVLIQYHPRLFQTIIQMTQNEDLRENMLRQVLEH
LSQQSESQYLKILTSLAEVATTNGHKLLSLSSNYDAQMKSLLRIVRMFCHVFRIGPSSPSNGIDMGYNGNKTPRSQVFKP
LELLWHSLDEWLVLIATELMKNKRDSTEITSILLKQKGQDQDAASIPPFEPPGPGSYENLSTGTRESKPDALAGRQEASA
DCQDVISMTANRLSAVIQAFYMCCSCQMPPGMTSPRFIEFVCKHDEVLKCFVNRNPKIIFDHFHFLLECPELMSRFMHI
IKAQPFKDRCEWFYEHLHSGQPDSDMVHRPVNENDILLVHRDSIFRSSCEVVSKANCAKLKQGIAVRFHGEEGMGQG
VVREWFDILSNEIVNPDYALFTQSADGTTFQPNSNSYVNPDHLNYFRFAGQILGLALNHRQLVNIYFTRSFYKHILGIPVN
YQDVASIDPEYAKNLQWILDNDISDLGLELTFSVETDVFGAMEEVPLKPGGGSILVTQNNKAEYVQLVTELRMTRAIQP
QINAFLQGFHMFIPPSLIQLFDEYELELLLSGMPEIDVSDWIKNTEYTSGYEREDPVIQWFWEVVEDITQEERVLLLQFVT
GSSRVPHGGFANIMGGSGLQNFTIAAVPYTPNLLPTSSTCINMLKLPEYPSKEILKDRLLVALHCGSYGYTMA

Isoform h (715 aa)

MSTFVIMKALQHHGQRDTAQILLLRGAKYLPDKNGVTPLDLCVQGGYGETCEVLIQYHPRLFQTIIQMTQNEDLRENM
LRQVLEHLSQQSESQYLKILTSLAEVATTNGHKLLSLSSNYDAQMKSLLRIVRMFCHVFRIGPSSPSNGIDMGYNGNKTP
RSQVFKPLELLWHSLDEWLVLIATELMKNKRDSTEITSILLKQKGQDQDAASIPPFEPPGPGSYENLSTGTRESKPDALAG
RQEASADCQDVISMTANRLSAVIQAFYMCCSCQMPPGMTSPRFIEFVCKHDEVLKCFVNRNPKIIFDHFHFLLECPELM
SRFMHIIKAQPFKDRCEWFYEHLHSGQPDSDMVHRPVNENDILLVHRDSIFRSSCEVVSKANCAKLKQGIAVRFHGEEG
MGQGVVREWFDILSNEIVNPDYALFTQSADGTTFQPNSNSYVNPDHLNYFRFAGQILGLALNHRQLVNIYFTRSFYKHI
LGIPVNYQDVASIDPEYAKNLQWILDNDISDLGLELTFSVETDVFGAMEEVPLKPGGGSILVTQNNKAEYVQLVTELRM
TRAIQPQINAFLQGFHMFIPPSLIQLFDEYELELLLSGMPEIDVSDWIKNTEYTSGYEREDPVIQWFWEVVEDITQEERVL
LLQFVTGSSRVPHGGFANIMGGSGLQNFTIAAVPYTPNLLPTSSTCINMLKLPEYPSKEILKDRLLVALHCGSYGYTMA
